# Enhancement of Self-Monitoring in a Web-Based Weight Loss Program by Extra Individualized Feedback and Reminders: Randomized Trial

**DOI:** 10.2196/jmir.4100

**Published:** 2016-04-12

**Authors:** Melinda Jane Hutchesson, Chor Yin Tan, Philip Morgan, Robin Callister, Clare Collins

**Affiliations:** ^1^ Priority Research Centre in Physical Activity and Nutrition, School of Health Sciences Faculty of Health and Medicine University of Newcastle Callaghan Australia; ^2^ School of Education Faculty of Education and Arts University of Newcastle Callaghan Australia; ^3^ School of Biomedical Sciences and Pharmacy Faculty of Health and Medicine University of Newcastle Callaghan Australia; ^4^ School of Health Sciences Faculty of Health and Medicine University of Newcastle Callaghan Australia

**Keywords:** weight loss, Web-based intervention, randomized controlled trial, commercial weight loss program, self-monitoring

## Abstract

**Background:**

Self-monitoring is an essential behavioral strategy for effective weight loss programs. Traditionally, self-monitoring has been achieved using paper-based records. However, technology is now more frequently used to deliver treatment programs to overweight and obese adults. Information technologies, such as the Internet and mobile phones, allow innovative intervention features to be incorporated into treatment that may facilitate greater adherence to self-monitoring processes, provide motivation for behavior change, and ultimately lead to greater weight loss success.

**Objective:**

The objective of our study was to determine whether the consistency of self-monitoring differed between participants randomly assigned to a basic or an enhanced 12-week commercial Web-based weight loss program.

**Methods:**

We randomly assigned a sample of 301 adults (mean age 42.3 years; body mass index 31.3 kg/m2; female 176/301, 58.5%) to the basic or enhanced group. The basic program included tools for self-monitoring (online food and exercise diary, and a weekly weigh-in log) with some feedback and reminders to weigh in (by text or email). The enhanced program included the basic components, as well as extra individualized feedback on self-monitoring entries and reminders (by text, email, or telephone) to engage with self-monitoring tools. We evaluated the level of self-monitoring by examining the consistency of self-monitoring of food, exercise, and weight during the 12 weeks. Consistency was defined as the number of weeks during which participants completed a criterion number of entries (ie, ≥3 days of online food or exercise diary records per week and ≥1 weigh-in per week).

**Results:**

The enhanced group’s consistency of use of self-monitoring tools was significantly greater than that of the basic group throughout the 12 weeks (median consistency for food 8 vs 3 weeks, respectively, *P*<.001; for exercise 2.5 vs 1 weeks, respectively, *P*=.003).

**Conclusions:**

Enhanced features, including additional individualized feedback and reminders, are effective in enhancing self-monitoring behaviors in a Web-based weight loss program.

**ClinicalTrial:**

Australian New Zealand Clinical Trials Registry (ANZCTR): ACTRN12610000197033; https://www.anzctr.org.au/Trial/Registration/TrialReview.aspx?id=335159 (Archived by WebCite at http://www.webcitation.org/6gCQdj21G)

## Introduction

Self-monitoring is a behavioral strategy considered essential for effective weight loss programs [1]. Self-monitoring typically involves systematic observation, measurement, and recording of dietary intake, exercise, and weight [1]. This monitoring may raise individuals’ awareness of their own actions, how and when these actions occur, and the initial and lasting impact on their actions [1]. It allows individuals to evaluate their progress toward goal attainment, reinforces behavior changes made, and highlights behaviors that may require further attention [2,3].

A systematic review evaluated the effects of self-monitoring of diet, exercise, and self-weighing on weight loss as part of a behavioral intervention program [4]. All 22 included studies supported the hypothesis that greater frequency of self-monitoring is associated with greater weight loss [4]. Due to the diversity of measurements of self-monitoring of dietary intake and exercise in the included studies, the reviewers could not determine an optimal frequency of self-monitoring of dietary intake and physical activity necessary for weight loss. However, the review concluded that individuals who weighed themselves at least once per week lost significantly more weight, which is consistent with an earlier systematic review [5]. Notably, the later review identified that very few studies have examined participants’ adherence to self-monitoring over time (ie, how consistently they self-monitored over time) and any association with weight loss [4].

Behavioral weight loss programs are traditionally delivered in a face-to-face format and self-monitoring is completed using paper-based diaries [6]. However, new treatment modalities using technologies, such as the Internet or mobile phone apps, have been developed. The multimedia capabilities of such technologies have the potential to minimize the obstacles associated with paper-based self-monitoring, such as reducing the participant’s burden by simplifying the recording process [7]. Furthermore, technology provides an opportunity for inclusion of features that may facilitate greater adherence to the self-monitoring process, enhance motivation for behavior change, and ultimately lead to greater weight loss success [4]. Such features include the provision of automated or tailored feedback on weight, dietary intake, or exercise levels or reminders (eg, text messages, emails) to complete program tasks such as self-monitoring [4,8]. Using meta-analysis, a recent systematic review demonstrated that eHealth weight loss programs with additional features achieved 1.46 kg greater weight loss postintervention than those providing a standard eHealth program alone [8]. However, few studies have investigated whether the provision of these additional self-monitoring-related features improves adherence to self-monitoring and facilitates greater weight loss. Burke et al [9] randomly assigned participants to self-monitor their dietary intake using 3 approaches: a personal digital assistant with no feedback; a personal digital assistant with daily tailored automated feedback; or a paper-based diary with no feedback. They found that after 24 months there was no significant difference in weight loss between the 3 groups, although the 2 personal digital assistant groups self-monitored on a significantly greater proportion of days than the paper-based monitoring group over the 24-month period [9]. This study highlighted the ability of technology to engage individuals in the self-monitoring process and, as the authors conclude, was an important “early step” in understanding how technology can be used for self-monitoring in weight loss programs. Further examination of whether specific components of technology-based weight loss programs can improve participants’ adherence to self-monitoring of diet, exercise, and weight is required.

We previously conducted a randomized controlled trial (RCT) comparing the efficacy of 2 versions (basic vs enhanced) of a commercial Web-based weight loss program for 12 weeks [10]. Both versions of the program included tools for self-monitoring (online food and exercise diary, and a weekly weigh-in log) with automated feedback on self-monitoring records and once-weekly reminders via text message or email to weigh in. The enhanced group also received additional weekly automated individualized feedback reports on current diet and exercise based on their previous week’s self-monitoring records, as well as extra reminders via text message, email, or phone to complete all self-monitoring records. Both groups lost weight; however, we found no significant difference in mean weight change between groups (basic -2.7, SD 4.0, enhanced -3.3, SD 4.5, *P*=.21) or the proportion of participants who achieved a clinically significant weight loss of 5% (basic 24.5%, enhanced 32.9%, *P*=.11) at 12 weeks based on intention-to-treat analysis [11].

Therefore, the aims of this investigation were to determine whether consistency (ie, number of weeks during which participants completed a criterion number of online entries) of self-monitoring of food intake, exercise, and weight differed between participants randomly assigned to the basic version and those assigned to the enhanced version of the commercial Web-based weight loss program, and whether the consistency of self-monitoring was related to weight loss after 12 weeks [10]. We hypothesized that the enhanced group would achieve significantly greater frequency and consistency of self-monitoring of dietary intake, exercise, and weight than the basic group.

## Methods

### Study Design

We collected data for this analysis as part of a commercial Web-based weight loss program RCT. The methods of the RCT have been published in detail elsewhere [10]. We investigated online self-monitoring behaviors in adults allocated to 1 of 2 versions of a commercial weight loss program with basic or enhanced features for 12 weeks.

### Participants and Recruitment

We recruited overweight and obese (body mass index, BMI, of 25 to 40 kg/m^2^) adults (18 to 60 years old) in the Hunter region of New South Wales, Australia, through media advertising (radio, TV, newspaper, flyers in general practitioner clinics, university website) from October to December 2009. To be included in the study, participants had to agree not to take part in other weight loss programs for the study duration; pass a health screening questionnaire; have access to a computer with Internet and an email account; and be able to attend assessment sessions at the University of Newcastle campus (Callaghan, Australia). Participants were ineligible for the study if they were pregnant or trying to conceive; had major medical illnesses; had physical inabilities such as orthopedic or joint problems; had lost 4.5 kg or more in the preceding 6 months; or were taking medications that affected or were affected by weight loss. We obtained written informed consent from all participants before their enrollment.

### Random Allocation to Groups

We initially randomly assigned participants to 1 of 3 groups (basic or enhanced treatment group, or a waiting list control) using a stratified randomized block design. Blocks of variable length (either 3 or 6) were used to stratify participants according to their sex and baseline BMI category (25 to <30; ≥30 to <35 or ≥35 to 40 kg/m^2^). After 12 weeks, we randomly reallocated participants in the control group to either the basic or enhanced group, using the same procedures. We analyzed the self-monitoring behaviors of all participants during their participation in the basic or enhanced group. Participants were informed of their group allocation in sealed envelopes, which contained their online program login details. Participants in the basic and enhanced groups and researchers assessing outcomes were blinded to participants’ assignment to treatment groups.

### Weight Loss Interventions

Participants were given free access to a basic or enhanced version of the commercial Web-based weight loss program The Biggest Loser Club [12] provided by SP Health Co (Sydney, NSW, Australia). The features of the Web-based program were designed based on social cognitive theory [13]. The program targeted the major factors of behavioral change, including self-efficacy, goal setting, self-monitoring, and social support. Both the basic and enhanced programs were conducted through the Web-based program for 12 weeks. Table 1 describes the key features of the basic and enhanced programs, specifically highlighting the self-monitoring tools, as well as features designed to encourage participants to self-monitor.

**Table 1:** Comparison of features of the basic and enhanced commercial Web-based weight loss programs.

	Basic and enhanced	Enhanced only
Self-monitoring tools	*Online food and exercise diary* to monitor energy intake and energy expenditure: participants were encouraged to self-monitor their dietary intake and exercise using an online diary at least 4 days per week. Participants recorded the type and amount of food or exercise by searching a database for the most appropriate item, selecting the appropriate measurement unit, and entering the amount. Participants *recorded weight* (weigh-in) as well as other body measurements (waist and hip girths) via website or text message, and were encouraged to record at least once per week.	No additional features were available.
Tools to enhance self-monitoring: feedback	*Online food and exercise diary:* Automated calculations of energy intake, energy expenditure, and energy balance were provided on the online diary page. Automated nutrition summaries were available via link on online diary page. Reported intake was compared with recommended nutrient targets for key nutrients: energy, total fats, saturated fat, protein, carbohydrate, sugars, fiber, sodium, calcium, iron, zinc, magnesium, iodine, selenium, vitamins B1, B2, B3, B6, B12, A, C, and folate (if entries made in online diary). * Weigh-ins:* entered weight data (and other measurements) were tracked and displayed graphically and in a body (body mass index) silhouette to demonstrate change over time.	A weekly automated individualized feedback report based on *online food and exercise diary* entries was provided via the website for the previous week. Feedback for key elements of diet and exercise (ie, weekly summary of energy intake and expenditure, saturated fat, fruit and vegetable intakes, frequency and intensity of physical activity, and time spent being active compared with national recommendations), usage patterns of the website (ie, cumulative average website visits, diary entries, and forum posts), and level of success with weight loss (ie, weight loss to date) was provided. The feedback used a color-coded traffic light system (green, amber, red) to indicate whether a participant was meeting recommendations (green), moving in the right direction (amber), or not meeting recommendations (red).
Tools to enhance self-monitoring: reminders	Participants were encouraged to *weigh-in* via once-weekly email or short message service text messaging reminders to enter weight on the website on the due date.	Weekly reminders to further motivate participants to log in to the website, *weigh in*, and use the *online food and exercise diary* were sent. The reminders escalated with urgency, starting with an initial reminder email, then a text message, and lastly a phone call if participants did not engage with the program. Reminders commenced when weigh-in was 2 days overdue or if no site visits were made in 3 days or site visits but no diary entries were made in 4 days.
Other tools	Participants set a weight loss goal and were assigned individualized daily calorie targets to facilitate a 0.5–1 kg weight loss per week (~2600 kJ less than their estimated energy requirements). Online education in the form of weekly tutorials, fact sheets, meal and exercise plans, and weekly challenges were provided. Access to weekly low-fat menu plan and grocery lists designed to meet nutrient reference values and assigned calorie target was available. Social support was available via online discussion forums.	An individualized weekly automated enrollment report based on responses to the enrollment survey was sent. It included an assessment of current weight and suggestions for appropriate weight loss goals; an energy balance assessment and recommended calorie target; an assessment of eating habits and behaviors, including saturated fat and fiber intake, daily servings of fruits and vegetables, high-risk eating behaviors (eg, skipping meals, not eating breakfast, drinking soft drinks), and nonhungry eating triggers; and weight loss motivation assessment.

### Measures

All self-monitoring data were collected by SP Health Co and provided to the researchers. Data stored by SP Health Co included the date a participant submitted a food exercise or weigh-in entry. To make a food or exercise entry, participants searched for and selected a food or exercise item from the database, selected a unit of measurement (eg, grams or cups for food, or minutes or distance in kilometers for exercise), and then recorded the amount. A weigh-in entry required a weight to be entered by participants either online or via text message. For the purposes of this study, we required a participant to make 1 entry per day (ie, enter 1 food item, 1 exercise, or 1 weight) for the day to be counted as self-monitoring and therefore be included in the calculation of consistency of self-monitoring.

Consistency of self-monitoring for this study refers to the number of weeks during which a criterion number of entries was made. For food and exercise the criterion number was ≥3 days of entries per week, as per the previous definition of Peterson et al [14]. For weigh-ins the criterion number was ≥1 weigh-in per week, as per previous systematic reviews indicating that individuals who weighed themselves at least once weekly lost significantly more weight [4,5].

To determine the relationship between categories of self-monitoring consistency and weight loss, we grouped participants into 3 levels of self-monitoring consistency over the 12 weeks, based on the number of weeks when they met the criterion number of self-monitoring entries (ie, ≥3 days of online food or exercise diary records per week and ≥1 weigh-in per week). The levels were defined as low consistency if participants met the criterion number of self-monitoring entries in ≤4 weeks; as moderate if they met the criterion between 5 and 8 weeks; or as high if they met the criterion for ≥9 weeks. Low, medium, and high were defined a priori, by dividing the number of intervention weeks (ie, 12 weeks) into 3 groups covering an equal number of weeks.

All other measurements were taken at the Human Performance Laboratory at the University of Newcastle, Callaghan Campus, with assessments at baseline and 12 weeks of the study. Height was measured to 0.1 cm using the stretch stature method on a Harpenden portable stadiometer (Holtain Limited, Dyfed, UK). Weight was measured with the participant wearing light clothing, without shoes, on a digital scale to 0.01 kg (model CH-150kp; A&D Mercury Pty Ltd, Adelaide, Australia). BMI was calculated as weight (kg)/height (m)^2^. Participants completed a survey at baseline that captured sociodemographic characteristics (age, sex, education level, ethnicity and income).

### Statistical Analysis

We analyzed the data using Stata 11.0 (StataCorp LP). Descriptive statistics are described as mean and SD for normally distributed continuous variables, median and interquartile range for nonnormal continuous data, and number (n) and percentage for categorical variables. We used chi square tests to compare the self-monitoring consistency each week (weeks 1 to 12) by treatment group. Due to the multiple comparisons, we applied the Bonferroni correction, with *P*<.004 (*P*=.05/12) considered statistically significant.

We also used chi-square tests to compare the total number of consistent weeks and consistency of self-monitoring groups (low, moderate, high) by treatment group. Analysis of variance tested for differences in percentage weight loss at 12 weeks between consistency of self-monitoring groups (low, moderate, high). We performed post hoc comparisons using the Tukey-Kramer method. An intention-to-treat approach was used for calculating percentage weight loss, with baseline observation carried forward for those lost to follow-up at 12 weeks. We considered *P*<.05 to be statistically significant for these single comparisons.

## Results

### Baseline Characteristics

Of the 591 adults who expressed interest in participating in the study, 309 were randomly assigned to 1 of 3 groups (basic n=99, enhanced n=106, waiting list control n=104). We randomly reassigned waiting list control group participants to a treatment group after 12 weeks (basic n=44, enhanced n=52, lost to follow-up n=8); therefore, this analysis included 301 participants (basic n=143, enhanced n=158). We previously reported participants’ characteristics at baseline [11]. In summary, 58.5% of participants were female (176/301), with a mean (SD) age of 42 (10.2) years, and most were born in Australia (273/301, 90.7%), were classified as obese (195/301, 64.8%), had an educational level higher than high school (210/301, 69.8%), and had a weekly household income of more than A$1500 (194/301, 64.5%). At baseline (treatment group entry), characteristics of the basic and enhanced group participants did not differ significantly [11], nor were there any differences between participants initially randomly assigned to the waiting list control group and their respective intervention groups.

### Attrition Rates

A total of 62 participants did not have their weight assessed at 12 weeks, resulting in 20.6% (62/301) attrition. There was no significant difference (*P*=.7) in attrition rates between the basic (31/143, 21.7%) and enhanced groups (31/158, 19.6%) at 12 weeks.

### Consistency of Self-monitoring

Table 2 describes the consistency of self-monitoring by treatment group. The median number of weeks during which participants made food and exercise entries on ≥3 days per week and weighed in once per week was significantly greater in the enhanced than in the basic group (food: 8 vs 3 weeks, respectively, *P*<.001; exercise: 2.5 vs 1 week, respectively, *P*=.003; weigh-ins: 11 vs 8 weeks, respectively, *P*<.001). The enhanced and basic groups differed significantly in the proportion of participants classified as having low, moderate, and high self-monitoring consistency for food (χ^2^_2_=18.9, *P*<.001), exercise (χ^2^_2_=10.0, *P*=.007) and weight (χ^2^_2_=16.5, *P*<.001) entries (Table 2).

**Table 2:** Frequency and level of consistency of self-monitoring^a^ by basic and enhanced groups of a commercial Web-based weight loss program over 12 weeks.

Data entered by participant		Basic (n=143)	Enhanced (n=158)	*P* value^b,c^
**Food**				
	No. of weeks ≥3 days of entries, median (IQR^d^)	3 (0–12)	8 (0–2)	<.001
	Low (n=133), n (%)	55.94 (80)	33.54 (53)	<.001
	Moderate (n=54), n (%)	18.18 (26)	17.72 (28)	
	High (n=114), n (%)	25.87 (37)	48.73 (77)	
**Exercise**				
	No. of weeks ≥3 days of entries, median (IQR)	1 (0–12)	2.5 (0–12)	.003
	Low (n=207), n (%)	77.62 (111)	60.76 (96)	.007
	Moderate (n=36), n (%)	8.39 (12)	15.19 (24)	
	High (n=58), n (%)	13.99 (20)	24.05 (38)	
**Weigh-ins**				
	No. weeks with 1 weigh-in, median (IQR)	8 (2–12)	11 (7–12)	<.001
	Low (n=79), n (%)	37.06 (53)	16.46 (26)	<.001
	Moderate (n=50), n (%)	13.99 (20)	18.99 (30)	
	High (n=172), n (%)	48.95 (70)	64.56 (102)	

^a^Levels were defined as low consistency if participants met the criterion number of self-monitoring entries in ≤4 weeks; as moderate if they met the criterion between 5 and 8 weeks; or as high if they met the criterion for ≥9 weeks.

^b^Wilcoxon test populations to compare between groups for median number of days, entries, and weeks with ≥3 days of entries.

^c^Chi square to compare between groups for number who used the self-monitoring feature, and number having low, moderate, or high consistency.

^d^IQR: interquartile range.

**Figure 1:**
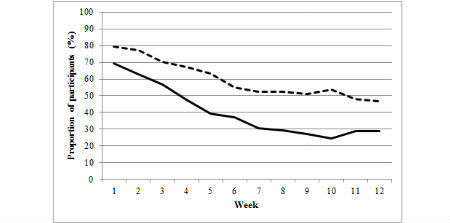
Proportion of participants from the basic (full line) and enhanced (dashed line) groups who consistently (3 or more days/week) made food entries to the online diary from weeks 1 to 12.

**Figure 2:**
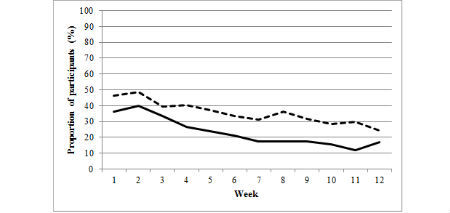
Proportion of participants from the basic (full line) and enhanced (dashed line) groups who consistently (3 or more days/week) made exercise entries to the online diary from weeks 1 to 12.

**Figure 3:**
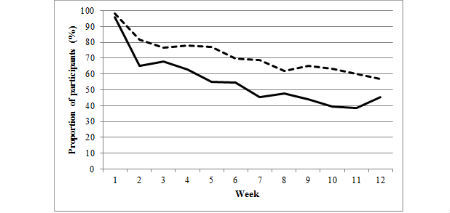
Proportion of participants from the basic (full line) and enhanced (dashed line) groups who consistently (1 or more days/week) weighed in from weeks 1 to 12.

The figures illustrate the proportion of participants from the basic and enhanced groups who consistently made an online diary food entry (Figure 1) or exercise entry (Figure 2) or weighed in (Figure 3) during each week of the program (weeks 1 to 12). A significantly higher proportion of the enhanced group than of the basic group made food entries ≥3 days per week from weeks 4 to 12 of the program. A significantly higher proportion of enhanced group participants than of basic group participants made exercise entries ≥3 days per week during weeks 8 and 11, and a significantly higher proportion of enhanced group participants than of basic group participants weighed in during weeks 2, 5, 7, 9, 10, and 11 (Multimedia Appendix 1). Notably, both the basic and enhanced groups’ consistency of self-monitoring declined from weeks 1 to 12 (Figure 1, Figure 2, Figure 3, and Multimedia Appendix 1). For example, in week 1 of the program, 79.6% (126/158) of enhanced group participants made food entries to the online diary ≥3 days compared with 46.8% (74/158) in week 12. In comparison, 69.2% (99/143) of basic group participants made food entries to the online diary in week 1 compared with 28.7% (41/143) in week 12.

### Consistency of Self-monitoring and Weight Loss

Consistency of self-monitoring strongly predicted weight loss. There was a significant difference in percentage weight loss by consistency of self-monitoring for food entries (*F*_2,298_=57.39, *P*<.001), exercise entries (*F*_2,298_=66.20, *P*<.001), and weigh-ins (*F*_2,298_=33.49, *P*<.001). Post hoc comparisons revealed that, for food and exercise entries to the online diary, participants whose consistency was high lost significantly more weight than those whose consistency was low or moderate, and those whose consistency was classified as moderate lost significantly more weight than those classified as low (food entries online diary for high: mean –6.2, SD 4.5%; moderate: –2.6, SD 3.7%; low –1.1, SD 3.0%; exercise entries online diary for high: mean –7.9, SD 4.7%; moderate –4.5, SD 3.8%; low: –1.7, SD 3.3%). For weigh-ins, participants whose consistency was high or moderate lost more weight (mean –4.9, SD 4.7% and –1.7, SD 3.0%, respectively) than those whose consistency was low (–0.7, SD 2.7%).

## Discussion

Our study found greater consistency of self-monitoring among participants randomly assigned to an enhanced version of a 12-week commercial Web-based weight loss program that included additional individualized feedback on self-monitoring records and reminders to engage with the self-monitoring tools. The 2 groups differed in weigh-ins, and in food and exercise entries to the online diary, although the enhanced features had the least impact on exercise entries. Greater consistency of self-monitoring of all 3 monitoring behaviors was associated with increased weight loss. However, there was no significant difference in weight loss between the enhanced and basic intervention groups.

The greater use of self-monitoring tools by the enhanced group may be associated with the push factors, such as the reminders, or the positive feedback or encouragement provided by the weekly feedback reports, which is supported by the research of Eysenbach [15].

Therefore, more in-depth evaluation of the multiple intervention components used in the enhanced program is warranted, to examine reasons for greater engagement with the self-monitoring tools by the enhanced group participants. Furthermore, the consistency of self-monitoring varied with the behavior (weight, food, or exercise) being monitored and decreased over the 12 weeks for all 3 behaviors in both groups. Self-monitoring entries of weight were much more consistent in both groups throughout the 12 weeks than either food or exercise entries. Reasons for this greater consistency of weight entries are not clear but possible explanations are the relative ease of providing weight entries and positive reinforcement of effort if weight continues to decline. The consistency of food entries was also reasonably high initially, particularly in the enhanced group, but decreased substantially over time, especially in the basic group. Consistency of exercise entries was relatively poor from week 1. Although a greater proportion of enhanced group participants consistently self-monitored exercise compared with the basic group over the 12 weeks, it was only during 2 weeks (weeks 8 and 11) that a higher proportion of enhanced group participants made exercise entries ≥3 days per week. The additional features (eg, reminders, feedback) provided in the enhanced intervention may not have prompted self-monitoring of exercise and suggests that a greater understanding of the barriers to self-monitoring of exercise is required. However, it is also possible that the poor consistency of exercise entries may be due to a lack of exercise being performed by participants.

There is evidence from systematic reviews that greater self-monitoring within weight loss interventions is associated with greater weight loss [4,5]. The provision of enhanced features within the Web-based weight loss program appears to have facilitated greater consistency of self-monitoring; however, this did not result in significantly greater weight loss in this study [11]. This is consistent with several recent studies that have been unable to demonstrate a significant difference in weight loss between Web-based interventions providing additional features, such as individualized feedback, online support groups, or behavioral lessons to facilitate greater weight loss success [16-19]. The similar rates of weight loss between the 2 groups in this study and in other studies [16-19] may have been due to both the standard and basic versions of these programs having included key features known to contribute to weight loss success (eg, goal setting, self-monitoring with some feedback, and social support). In this study, although the enhanced features motivated more participants to use the self-monitoring tools, the additional feedback provided may not have assisted all participants to adequately self-regulate their behaviors in order to lose weight. For example, the extra feedback provided was individualized to behaviors reported by the participants as part of their self-monitoring records. Therefore, if they reported the same behavior each week, they would continue to receive an identical feedback message in subsequent weekly feedback reports. Furthermore, although the enhanced group’s use of the self-monitoring tools was significantly higher than that of the basic group, the use of the tools varied among study participants in both groups, as evidenced by the large interquartile ranges reported for all self-monitoring metrics. Clearly not all study participants were motivated to self-monitor by the enhanced program features, nor did all participants require the enhanced features to facilitate adequate levels of self-monitoring. Notably, both groups demonstrated declining rates of self-monitoring over time, which is consistent with previous reports of the use of online public health interventions [20] and Web-based weight loss programs (eg, [16,21]), including the program in this study [22]. This may have further contributed to the nonsignificant difference in weight loss between the 2 groups.

In a cohort study, we previously identified that a higher median number of days when participants used the self-monitoring features of the basic version of the program was associated with significantly greater weight loss [23]. A limitation of that study was that it relied on self-reported weight data. The results of the RCT reported here, with objective assessment of weight and use of self-monitoring tools, confirm our previous findings [23]. Participants who were highly consistent (≥9 weeks out of 12) in self-monitoring weight (≥1 day/week) and in reporting food or exercise in the online diary (≥3 days/week) lost significantly more weight (5% to 8%). Our findings are supported by Krukowski et al [24], who demonstrated that participants who consistently self-monitored during a 6-month online behavioral weight control program were significantly more likely to achieve clinically important weight loss. They found that participants who self-monitored on ≥6 days during the initial stages of the program (weeks 1 to 4) were more likely to achieve clinically important weight loss after 6 months, as well as those who self-monitored during the later weeks of the intervention (weeks 9 to 24) [24]. Further experimental research is required to determine whether the association between self-monitoring consistency and weight loss observed in this study is indicative of a cause-effect relationship between self-monitoring and weight loss success.

### Study Strengths and Limitations

A strength of this RCT was the large sample including substantial proportions of both male and female participants, and use of objective measures of self-monitoring. Potential limitations include that we evaluated self-monitoring behaviors during the 12-week weight loss intervention. Therefore, we do not know whether self-monitoring was continued or maintained beyond the 12-week period, and whether this was associated with further weight loss. This is an area where future research is warranted, as it was recently suggested that frequency and consistency of self-monitoring of dietary intake after a 6-month weight loss program improved weight loss success at 12 months [14]. Our study focused on 1 behavioral strategy (self-monitoring) within the commercial weight loss program, and did not consider the potential influence of other key behavioral strategies (eg, social support via the discussion forum, or provision of tailored feedback via automated feedback reports). Due to errors with tracking of participant’s usage of other program components during the trial, this analysis is not possible. The definition of consistency of self-monitoring was based on previous literature where possible; however, as no universally accepted metrics for self-monitoring exist, the results may vary with the use of different cut points.

### Conclusion

Enhanced program features, such as reminders and tailored feedback, facilitated greater consistency of self-monitoring of food, exercise, and weight during a 12-week commercial Web-based weight loss program. However, there were no significant differences in weight loss between the enhanced and basic intervention groups. Given the strong association between self-monitoring of these behaviors and successful weight change outcomes, further evaluation of individuals’ experiences with self-monitoring and intervention components designed to promote self-monitoring (ie, reminders, tailored feedback) is warranted. This will provide greater insight into factors contributing to group and individual variations in engagement with self-monitoring tools and facilitate the design of Web-based weight loss interventions that are adaptive and provide individually tailored features to optimize self-monitoring (eg, frequency and mode of reminders, language used in feedback).

## Appendix

Multimedia Appendix 1 Consistency of use of the commercial web-based weight loss program's self-monitoring tools (online diary to monitor food and exercise and weigh-ins) by treatment group (basic vs enhanced) from weeks 1 to 12.

## Abbreviations

BMI: body mass index
RCT: randomized controlled trial

## References

[1] Foster GD, Makris AP, Bailer BA (2005). Behavioral treatment of obesity. Am J Clin Nutr.

[2] Kirschenbaum DS (1987). Self-regulatory failure: a review with clinical implications. Clin Psychol Rev.

[3] Baker RC, Kirschenbaum DS (1993). Self-monitoring may be necessary for successful weight control. Behav Ther.

[4] Burke LE, Wang J, Sevick MA (2011). Self-monitoring in weight loss: a systematic review of the literature. J Am Diet Assoc.

[5] Vanwormer JJ, French SA, Pereira MA, Welsh EM (2008). The impact of regular self-weighing on weight management: a systematic literature review. Int J Behav Nutr Phys Act.

[6] Wadden TA, Webb VL, Moran CH, Bailer BA (2012). Lifestyle modification for obesity: new developments in diet, physical activity, and behavior therapy. Circulation.

[7] Illner A, Freisling H, Boeing H, Huybrechts I, Crispim SP, Slimani N (2012). Review and evaluation of innovative technologies for measuring diet in nutritional epidemiology. Int J Epidemiol.

[8] Hutchesson M, Rollo ME, Krukowski R, Ells L, Harvey J, Morgan PJ, Callister R, Plotnikoff R, Collins CE (2015). eHealth interventions for the prevention and treatment of overweight and obesity in adults: a systematic review with meta-analysis. Obes Rev.

[9] Burke LE, Styn MA, Sereika SM, Conroy MB, Ye L, Glanz K, Sevick MA, Ewing LJ (2012). Using mHealth technology to enhance self-monitoring for weight loss: a randomized trial. Am J Prev Med.

[10] Collins CE, Morgan PJ, Jones P, Fletcher K, Martin J, Aguiar EJ, Lucas A, Neve M, McElduff P, Callister R (2010). Evaluation of a commercial web-based weight loss and weight loss maintenance program in overweight and obese adults: a randomized controlled trial. BMC Public Health.

[11] Collins CE, Morgan PJ, Hutchesson MJ, Callister R (2013). Efficacy of standard versus enhanced features in a Web-based commercial weight-loss program for obese adults, part 2: randomized controlled trial. J Med Internet Res.

[12] SP Health The Biggest Loser Club.

[13] Bandura A (1986). Social Foundations of Thought and Action: A Social Cognitive Theory.

[14] Peterson ND, Middleton KR, Nackers LM, Medina KE, Milsom VA, Perri MG (2014). Dietary self-monitoring and long-term success with weight management. Obesity (Silver Spring).

[15] Eysenbach G (2005). The law of attrition. J Med Internet Res.

[16] Brindal E, Freyne J, Saunders I, Berkovsky S, Smith G, Noakes M (2012). Features predicting weight loss in overweight or obese participants in a web-based intervention: randomized trial. J Med Internet Res.

[17] Hersey JC, Khavjou O, Strange LB, Atkinson RL, Blair SN, Campbell S, Hobbs CL, Kelly B, Fitzgerald TM, Kish-Doto J, Koch MA, Munoz B, Peele E, Stockdale J, Augustine C, Mitchell G, Arday D, Kugler J, Dorn P, Ellzy J, Julian R, Grissom J, Britt M (2012). The efficacy and cost-effectiveness of a community weight management intervention: a randomized controlled trial of the health weight management demonstration. Prev Med.

[18] Pullen CH, Hageman PA, Boeckner L, Walker SN, Oberdorfer MK (2008). Feasibility of Internet-delivered weight loss interventions among rural women ages 50-69. J Geriatr Phys Ther.

[19] Wing RR, Crane MM, Thomas JG, Kumar R, Weinberg B (2010). Improving weight loss outcomes of community interventions by incorporating behavioral strategies. Am J Public Health.

[20] Glasgow RE (2007). eHealth evaluation and dissemination research. Am J Prev Med.

[21] Ware LJ, Hurling R, Bataveljic O, Fairley BW, Hurst TL, Murray P, Rennie KL, Tomkins CE, Finn A, Cobain MR, Pearson DA, Foreyt JP (2008). Rates and determinants of uptake and use of an internet physical activity and weight management program in office and manufacturing work sites in England: cohort study. J Med Internet Res.

[22] Neve MJ, Collins CE, Morgan PJ (2010). Dropout, nonusage attrition, and pretreatment predictors of nonusage attrition in a commercial Web-based weight loss program. J Med Internet Res.

[23] Neve M, Morgan PJ, Collins CE (2011). Weight change in a commercial web-based weight loss program and its association with website use: cohort study. J Med Internet Res.

[24] Krukowski RA, Harvey-Berino J, Bursac Z, Ashikaga T, West DS (2013). Patterns of success: online self-monitoring in a web-based behavioral weight control program. Health Psychol.

