# Supplementary material for: Enhancement of Self-Monitoring in a Web-Based Weight Loss Program by Extra Individualized Feedback and Reminders: Randomized Trial
Source: J Med Internet Res. 2016 Apr 12;18(4):e82. doi: 10.2196/jmir.4100 (PMC4846785; doi:10.2196/jmir.4100)
Supplement: Multimedia Appendix 1 [file jmir_v18i4e82_app1.pdf]

Multimedia Appendix 1

Table M1: Consistency of use of the commercial web-based weight loss programs self-monitoring tools (online diary to monitor food and exercise and weigh-ins) by treatment group (basic vs. enhanced) from Week 1 to 12

**Week**	**Basic**	**Enhanced**	**Pearson chi-squared**	**P value**
Food entries to online diary				
**Compliance: Proportion who used ≥3 days/week (%)**				
1	69.2	79.6	4.40	.036
2	62.9	77.2	7.35	.007
3	56.6	70.3	6.02	.014
4	47.6	67.1	11.75	.001
5	39.2	63.3	17.51	<.001
6	37.1	55.1	9.78	.002
7	30.8	52.5	14.58	<.001
8	29.4	52.5	16.58	<.001
9	27.3	51.3	18.03	<.001
10	24.5	53.8	26.92	<.001
11	28.7	48.1	11.93	.001
12	28.7	46.8	10.49	.001
Exercise entries to online diary				
**Compliance: Proportion who used ≥3 days/week (%)**				
1	36.4	46.2	2.99	.084
2	39.9	48.7	2.39	.122
3	33.6	39.2	1.04	.307
4	26.6	40.5	6.50	.011
5	23.8	37.3	6.47	.011
6	21.0	33.5	5.93	.015
7	17.5	31.0	7.41	.016
8	17.5	36.1	13.09	<.001
9	17.5	31.7	18.05	.005
10	15.4	28.5	7.44	.006
11	11.9	29.8	14.30	<.001
12	16.8	24.1	2.42	.119
Weigh-ins				
**Compliance: Proportion who used 1 day/week (%)**				
1	95.8	98.1	1.37	.24
2	65.0	81.7	10.70	.001
3	67.8	76.6	2.88	.09
4	62.9	77.9	8.07	.01
5	55.2	77.2	16.33	<.001
6	54.6	69.6	7.28	.01
7	45.5	69.0	17.04	<.001
8	47.6	62.0	6.36	.01
9	44.1	65.2	13.55	<.001
10	39.2	63.3	17.51	<.001
11	38.5	60.1	14.09	<.001
12	45.5	57.0	3.98	.046

Chi-squared test X^2 (2, 301) to compare the number of participants per group who used the self-monitoring tools
